# Mechanistic insight into the photodynamic effect mediated by porphyrin-fullerene C_60_ dyads in solution and in *Staphylococcus aureus* cells†

**DOI:** 10.1039/c8ra04562c

**Published:** 2018-06-21

**Authors:** M. Belén Ballatore, Mariana B. Spesia, M. Elisa Milanesio, Edgardo N. Durantini

**Affiliations:** Departamento de Química, Facultad de Ciencias Exactas, Físico-Químicas y Naturales, Universidad Nacional de Río Cuarto Ruta Nacional 36 Km 601, X5804BYA Río Cuarto Córdoba Argentina edurantini@exa.unrc.edu.ar +54 358 4676157

## Abstract

The photodynamic action mechanism sensitized by a non-charged porphyrin-fullerene C_60_ dyad (TCP-C_60_) and its tetracationic analogue (TCP-C_60_^4+^) was investigated in solution and in *Staphylococcus aureus* cells. The ability of both dyads to form a photoinduced charge-separated state was evidenced by the reduction of methyl viologen in *N*,*N*-dimethylformamide (DMF). Moreover, the formation of superoxide anion radicals induced by these dyads was detected by the reduction of nitro blue tetrazolium. Also, photosensitized decomposition of l-tryptophan (Trp) was investigated in the presence of reactive oxygen species (ROS) scavengers. The addition of β-carotene and sodium azide had a slight effect on reaction rate. However, photooxidation of Trp mediated by TCP-C_60_ was negligible in the presence of d-mannitol, while no protection was found using TCP-C_60_^4+^. In a polar medium, these dyads mainly act by a contribution of type I pathway with low generation of singlet molecular oxygen, O_2_(^1^Δ_g_). In *S. aureus* cell suspensions, an aerobic atmosphere was required for the photokilling of this bacterium. The photocytotoxicity induced by TCP-C_60_ was increased in D_2_O with respect to water, while a small effect was found using TCP-C_60_^4+^. Furthermore, photoinactivation of microbial cells was negligible in the presence of sodium azide. The addition of d-mannitol did not affect the photoinactivation induced by TCP-C_60_. In contrast, *S. aureus* cells were protected by d-mannitol when TCP-C_60_^4+^ was used as a photosensitizer. Also, generation of O_2_(^1^Δ_g_) in the *S. aureus* cells was higher for TCP-C_60_ than TCP-C_60_^4+^. Therefore, TCP-C_60_ appears to act in microbial cells mainly through the mediation of O_2_(^1^Δ_g_). Although, a contribution of the type I mechanism was found for cell death induced by TCP-C_60_^4+^. Therefore, these dyads with high capacity to produce photoinduced charge-separated state represent interesting photosensitizers to inactivate microorganisms by type I or type II mechanisms. In particular, TCP-C_60_ may be located in a non-polar microenvironment in the cells favoring a type II pathway, while a contribution of the type I mechanism was produced using the cationic TCP-C_60_^4+^.

## Abstract 2

The photodynamic action mechanism sensitized by a non-charged porphyrin-fullerene C_60_ dyad and its tetracationic analogue was investigated in solution and in *Staphylococcus aureus* cells.

## Graphical abstract

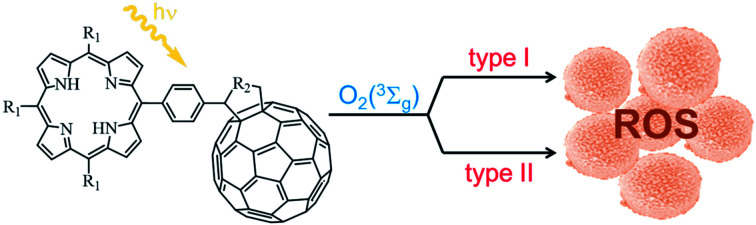

## 1. Introduction

Nowadays, infections caused by bacteria are becoming difficult to eradicate. This is mainly due to the continuous emergence of multidrug resistant strains.^1,2^ Thus, infections that could be treated without problem in the past, today they have become a challenge to be eradicated in hospitals throughout the world.^3–6^ Therefore, new treatments to deal with this situation are being sought.^7,8^ A new alternative to controlling microorganism infections is photodynamic inactivation (PDI).^9^ This therapy is based on the addition of a photosensitizer that rapidly binds to microbial cells. Excitation of the photosensitizer with light of an appropriate wavelength in the presence of molecular oxygen in the ground state, O_2_(^3^Σ_g_), produces reactive oxygen species (ROS).^10^ In PDI, two mechanisms can be mainly involved after activation of the photosensitizer.^11^ In the type I pathway, the photosensitizer excited triplet state can react with different substrates by electron or proton transfer to form free radicals. These radicals can also interact with O_2_(^3^Σ_g_) producing ROS, such as superoxide anion radical (O_2_˙^−^), hydroxyl radical (HO˙) and hydrogen peroxide (H_2_O_2_). In the type II partway, the photosensitizer generates singlet molecular oxygen, O_2_(^1^Δ_g_), by energy transfer. Thus, generation of ROS can simultaneously occur through type I and type II mechanisms. The ROS generated can rapidly react with a variety of substrates in microorganism cells inducing damage in biomolecules.^12^ Thus, these changes yield a loss of biological functionality leading to cell inactivation.

**Fig. 1:**
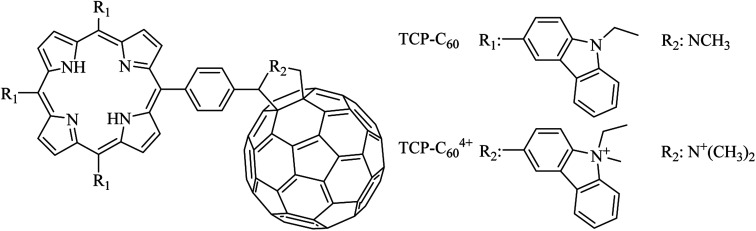
Molecular structures of TCP-C_60_ and TCP-C_60_^4+^.

In recent years, varieties of molecules have been proposed as photosensitizers. The design and synthesis of new compounds is still developing in order to find photosensitizers able to efficiently inactivate different bacterial strains in low concentration and in a shorter period of time.^13^ In particular, porphyrin and fullerene C_60_ derivatives were investigated as effective compounds to eradicate Gram-positive and Gram-negative bacteria after illumination with visible light.^14,15^ In a previous work, a novel porphyrin-fullerene C_60_ dyad (TCP-C_60_) was synthetized with three carbazoyl groups attached to the tetrapyrrolic macrocycle at the *meso* positions (Fig. 1).^16^ The three carbazoles and the pyrrolidinium ring, which links porphyrin covalently to fullerene C_60_, were positively charged by exhaustive methylation to produce a tetracationic photosensitizer (TCP-C_60_^4+^, Fig. 1). The photoinactivation ability of these neutral and cationic dyads was for first time investigated in *Staphylococcus aureus*. This microorganism was chosen because its ability to acquire resistance to antibiotics.^17,18^ Antibiotic resistant *S. aureus* is endemic in hospitals worldwide and it causes substantial morbidity and mortality.^19^ Ballatore *et al.* demonstrated for first time that TCP-C_60_ and TCP-C_60_^4+^ are efficient photosensitizers to inactivate *S. aureus*.^16^ Therefore, in the present work we are interested in to obtain mechanistic insight about photodynamic processes involved in the inactivation of *S. aureus* cells mediated by TCP-C_60_ and TCP-C_60_^4+^. First, photodynamic properties were investigated in solution to obtain evidence about the formation of charge separation state and production of ROS. After that, photoinactivation of *S. aureus* was studied under different conditions, varying the medium of cell suspensions and using specific scavengers of ROS. These experiments were used to increase the knowledge of the balance between type I and type II mechanisms sensitized by porphyrin-C_60_ dyads in a Gram-positive bacterium.

## 2. Experimental

### 2.1. General

Absorption spectra were performed on a Shimadzu UV-2401PC spectrometer (Shimadzu Corporation, Tokyo, Japan). Fluorescence spectra were carried out on a Spex FluoroMax spectrofluorometer (Horiba Jobin Yvon Inc, Edison, NJ, USA). Spectra were achieved using a quartz cell of 1 cm path length. Fluence rates were measured with a Radiometer Laser Mate-Q (Coherent, Santa Clara, CA, USA). Solutions were irradiated using a Cole-Parmer illuminator 41 720-series (Cole-Parmer, Vernon Hills, IL, USA) with a 150 W halogen lamp through a high intensity grating monochromator (Photon Technology Instrument, Birmingham, NJ, USA) with a fluence rate of 0.36 mW cm^−2^ at 428 nm, 0.38 mW cm^−2^ at 434 nm and 0.50 mW cm^−2^ at 562 nm. Optical filters (GG455 cutoff filter) were used to select a wavelength range between 455 and 800 nm (30 mW cm^−2^). Samples were irradiated in 1 cm path length quartz cells containing 2 mL of solution at 25.0 ± 0.5 °C. Cell growth was determined by absorption with a Turner SP-830 spectrophotometer (Dubuque, IA, USA). The visible light source to irradiate cell suspensions was a Novamat 130 AF (Braun Photo Technik, Nürnberg, Germany) slide projector with a 150 W lamp. A 2.5 cm glass cuvette filled with water was used to remove the heat from the lamp. A wavelength range between 350 and 800 nm was selected by optical filters with a fluence rate of 90 mW cm^−2^. Chemicals from Aldrich (Milwaukee, WI, USA) and solvents (GR grade) from Merck (Darmstadt, Germany) were used without further purification.

### 2.2. Photosensitizers

5,10,15,20-Tetrakis[3-(*N*-ethylcarbazoyl)]porphyrin (TCP), TCP-C_60_ and TCP-C_60_^4+^ were synthesized as previously described.^16^ Stock solution of 0.5 mM photosensitizer were prepared by dissolution in 1 mL of *N*,*N*-dimethylformamide (DMF). The concentration was checked by absorption, considering the value of molar extinction coefficient at Soret band in DMF (*ε* = 2.81 × 10^5^ M^−1^ cm^−1^ at 434 nm for TCP, *ε* = 2.52 × 10^5^ M^−1^ cm^−1^ at 431 nm for TCP-C_60_, *ε* = 2.49 × 10^5^ M^−1^ cm^−1^ at 432 nm for TCP-C_60_^4+^).^16^

### 2.3. Reduction of methyl viologen (MV^2+^)

Solutions of dyad (3.0 μM), MV^2+^ (0.8 mM) and *N*,*N*,*N′*,*N′*-tetramethyl-1,1-naphthidine (TMN, 0.4 mM) in DMF/5% water were irradiated with light at *λ*_irr_ = 433 nm under an argon atmosphere. The photochemical reaction was monitored by following the increase of the absorbance at *λ* = 398 nm and *λ* = 606 nm.^20^

### 2.4. Photooxidation of nitro blue tetrazolium (NBT)

Solutions of dyad (1.2 μM), NBT (0.2 mM) and NADH (0.5 mM) in DMF/10% water were irradiated with light at *λ*_irr_ = 428 nm. Control experiments were performed in absence of one of the substrates (NBT or NADH) and with both substrates without the dyad. The progress of the reaction was monitored by following the increase of the absorbance at *λ* = 560 nm.^21^

### 2.5. Photooxidation and singlet excited state deactivation of tryptophan (Trp)

Solutions of dyad (absorbance 0.1 at Soret band) and Trp (20 μM) in DMF were irradiated with light at *λ*_irr_ = 428 nm. Photooxidation of Trp was determined by the decrease of the fluorescence intensity (I) at *λ* = 340 nm, exciting the samples at *λ*_exc_ = 290 nm. Also, decomposition of Trp by dyads was investigated by adding β-carotene (5.6 μM) in DMF, sodium azide (50 mM) in DMF/2.5% water and d-mannitol (50 mM) in DMF/5% water. Solutions of Trp and dyad in the presence of different scavengers of ROS were irradiated with light at *λ*_irr_ = 562 nm. Under these conditions, the fluorescence intensity correlates linearly with Trp concentrations. The observed rate constants (*k*_obs_) were calculated by a linear least-squares fit of semi-logarithmic plots of ln(*I*_0_/*I*) *vs.* time.^21^ Singlet excited state deactivation of Trp by photosensitizer was investigated using the Stern–Volmer's eqn (1).^22^1
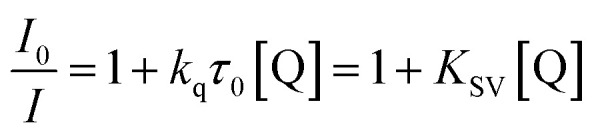
where *I*_0_ and *I* are the fluorescence intensity of Trp in the absence and in the presence of quencher, *k*_q_ represents the biomolecule quenching rate constant, *τ*_0_ the excited state lifetime of Trp in the absence of photosensitizer, [Q] is the photosensitizer concentration and *K*_SV_ is the Stern–Volmer quenching constants.

### 2.6. Bacterial strain and preparation of cultures

The microorganism used in this study was the reference strain *S. aureus* ATCC 25923.^16^ This bacterium was grown in a rotator shaker (100 rpm) at 37 °C in tryptic soy (TS, Britain, Buenos Aires, Argentina) broth overnight. Aliquots (∼60 μL) of this culture were aseptically transferred to 4 mL of fresh medium and incubated with agitation at 37 °C to middle of the logarithmic phase (absorbance ∼0.3 at 660 nm). Cells were centrifuged (3000 rpm for 15 min) and re-suspended in equal amount of 10 mM phosphate-buffered saline (PBS, pH 7.2) solution. Then the cells were diluted 1/1000 in PBS, corresponding to ∼10^6^ colony forming units (CFU) mL^−1^. In all the experiments, 2 mL of the cell suspensions in Pyrex brand culture tubes (13 × 100 mm) were used. Cell suspensions were serially diluted with PBS and each solution was quantified by using the spread plate technique in triplicate. Viable bacteria were monitored and the number of CFU ml^−1^ was determined on TS agar plates after ∼24 h incubation at 37 °C.

### 2.7. Photoinactivation of bacterial cell suspensions

Cell suspensions of *S. aureus* (2 mL, ∼10^6^ CFU mL^−1^) in PBS were incubated with 1 μM dyad in the dark at 37 °C for 30 min with agitation (100 rpm). The dyad was added from a stock solution 0.5 mM in DMF. After that, the cultures were exposed to visible light for 15 min. Studies in anoxic conditions were performed by displacing the oxygen with argon in the cultures flasks for 15 min before irradiation and keeping an argon atmosphere during the experiments. For photoinactivation assays in D_2_O, cells were centrifuged (3000 rpm for 15 min) and re-suspended in 2 mL PBS in D_2_O. Then the cell suspensions were incubated with 1 μM dyad as described above. Sodium azide or d-mannitol was added to bacterial suspensions from stock solutions in water (2 M and 1 M, respectively). Cell suspensions were incubated with 50 mM sodium azide or d-mannitol for 30 min at 37 °C in dark before the treatment with 1 μM dyad, as previously described.^23^

### 2.8. Steady state photolysis in bacterial cell suspensions

Cell suspensions of *S. aureus* (2 mL, ∼10^6^ CFU mL^−1^) were treated with 10 μM 9,10-dimethylanthracene (DMA) for 30 min in dark at 37 °C. Cells were harvested by centrifugation (3000 rpm for 15 min) and re-suspended in 2 mL PBS. Then, cells were incubated with 1 μM dyad for 30 min in dark at 37 °C. Samples (2 mL) were irradiated in 1 cm path length quartz cells with visible light 455–800 nm. Photooxidation of DMA was determined by following the decrease in the fluorescence intensity at *λ* = 427 nm, exciting the samples at *λ*_exc_ = 378 nm. In these conditions, the fluorescence intensity correlates linearly with DMA concentrations. The values of *k*_obs_ were obtained by a linear least squares fit of characteristics plots of ln(*I*_0_/*I*) *vs.* time.^23^

### 2.9. Controls and statistical analysis

Control experiments of *S. aureus* cultures were carried out with and without dyad in the dark and without dyad with irradiated cells. The amount of DMF (<1% v/v) used in each experiment was not toxic to bacterial cells. Three values were obtained per each condition and each experiment was repeated separately three times. The significance of the PDI effect of each dyad on microbial cells viability was assessed by one-way analysis of variance (ANOVA). A *p*-value below 0.05 was considered statistically significant. Data were represented as the mean ± standard deviation of each group.

## 3. Results and discussion

### 3.1. Photosensitized reduction of MV^2+^

To test the electron transfer ability of TCP-C_60_ and TCP-C_60_^4+^, the photoreduction of MV^2+^ in presence of TMN was studied in DMF/5% water. In these experiments, MV^2+^ can act as an electron acceptor.^20^ A comparison between the electron reduction potential of C_60_ moiety (−0.66 V) and MV^2+^ (−0.44 V) indicated that an electron transfer from C_60_ to MV^2+^ is exothermic by 0.22 eV.^20,24^ Moreover, TMN can be used as an electron donor. Taking into account the oxidation potential of TCP moiety (0.67 V) and the external electron donor TMN (0.43 V), electron transfer from TMN to TCP structure is expected to be exothermic by 0.24 eV.^20,24^ Therefore, the formation of a photoinduced charge-separated state (TCP˙^+^-C_60_˙^−^) can be accompanied by the reduction of MV^2+^ and oxidation of TMN.

**Fig. 2:**
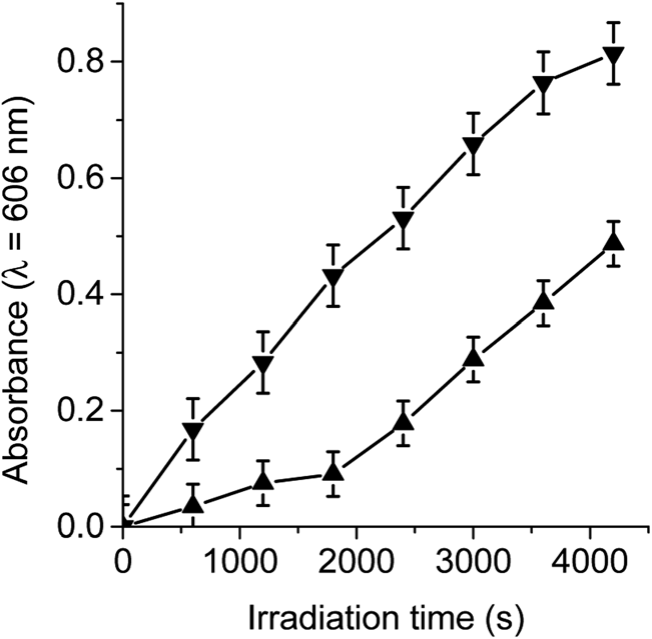
Time course of MV^2+^ (0.8 mM) reduction as an increase in the absorption at *λ* = 606 nm sensitized by TCP-C_60_ (▲) (3.0 μM) and TCP-C_60_^4+^ (▼) (3.0 μM) containing TMN (0.4 mM) after 10 min irradiation periods with light at *λ*_irr_ = 433 nm under an argon atmosphere in DMF/5% water.

Under an argon atmosphere, the photoinduced charge-separated state (TCP˙^+^-C_60_˙^−^) can be formed by irradiation with light at 433 nm. This wavelength was used because the absorption spectra of TCP-C_60_ and TCP-C_60_^4+^ showed a Soret band at 433 nm due to the free base porphyrin unit.^16^ When dyad/MV^2+^/TMN system was irradiated, a progressive increase in the characteristic absorption bands of MV˙^+^ was noted at 398 and 606 nm (Fig. S1†). The results after different irradiation periods are shown in Fig. 2, monitoring the generation of MV˙^+^ at 606 nm. Thus, the reduction of MV^2+^ and the oxidation of TMN were accomplished by the oxidation of TCP˙^+^ and reaction of C_60_˙^−^ in the dyad, respectively. By contrast, no reaction occurs in the dark or in the absence of the dyad under illumination. Moreover, it is important to note that no reaction occurs when irradiating the dyad with the electron acceptor or with the electron donor (results do not show). Thereby, both dyads are capable of forming a photoinduced charge-separated state in a polar medium as DMF/5% water. However, the MV^2+^ reduction by TCP-C_60_ was significantly slower than that by TCP-C_60_^4+^ (Fig. 2). This difference may be due to the partial aggregation of non-charged TCP-C_60_ by the addition of water to the DMF solution. On the other hand, in the presence of O_2_(^3^Σ_g_) the reduction of MV^2+^ was not observed because the oxygen catalyzed the back electron transfer process between C_60_˙^−^ and TCP˙^+^ (Video S1†).^25^

It was previously demonstrated that TCP-C_60_ and TCP-C_60_^4+^ have a very weak emission at ∼667 nm from the porphyrin moiety in DMF, with fluorescence quantum yield of *Φ*_F_ ∼ 4 × 10^−3^.^16^ The quenching efficiencies (*ɳ*_q_) of the porphyrin excited singlet state by the attached fullerene moiety were estimated to be *ɳ*_q_ > 0.96. These results are in accordance with the formation of a photoinduced charge-separated state (TCP˙^+^-C_60_˙^−^). Also, a value of 0.62 eV was obtained for the driving force for the initial charge separation from ^1^TCP* to C_60_.^16^ Similar results were previously observed with a porphyrin-fullerene C_60_ dyad metallated with Zn(ii).^20^ In this case, the efficiency of charge transfer was favoured by the presence of the metal. Fukuzumi *et al.* demonstrated that the generated C_60_˙^−^ moiety in ZnP˙^+^-C_60_˙^−^ dyad and in ZnP˙^+^-H_2_P-C_60_˙^−^ triad undergoes one electron oxidation by hexyl viologen (HV^2+^), whereas the ZnP˙^+^ moiety was reduced by a NADH analogues, 1-benzyl-1,4-dihydronicotinamide and 10-methyl-9,10-dihydroacridine.^26^ Thus, ZnP-C_60_ and ZnP-H_2_P-C_60_ donor–acceptor ensembles act in benzonitrile as efficient photocatalysts for the uphill oxidation of NADH analogues by HV^2+^. On the other hand, Dallas *et al.* demonstrated the intramolecular charge transfer between pyrene donor and fullerene C_60_/C_70_ acceptors, which showed the ability of these molecules to accept electrons.^27^ Moreover, Wang *et al.* prepared a complex micelle from zinc tetrakis(4-sulfonatophenyl)porphyrin (ZnTPPS), modified fullerene (mC_60_) and poly(ethyleneglycol)-*block*-poly(l-lysine) (PEG-*b*-PLys) by electrostatic interactions. The complex micelle exhibited high electron transfer performance in the photocatalytic reduction of MV^2+^. In the micellar core, ZnTPPS and mC_60_ molecules are surrounded by each other which ensured effective energy migration from the donor to the acceptor.^28^ Therefore, porphyrin-fullerene C_60_ dyads are effective molecular systems to form photoinduced charge-separated state.

### 3.2. Photodecomposition of NBT

**Fig. 3:**
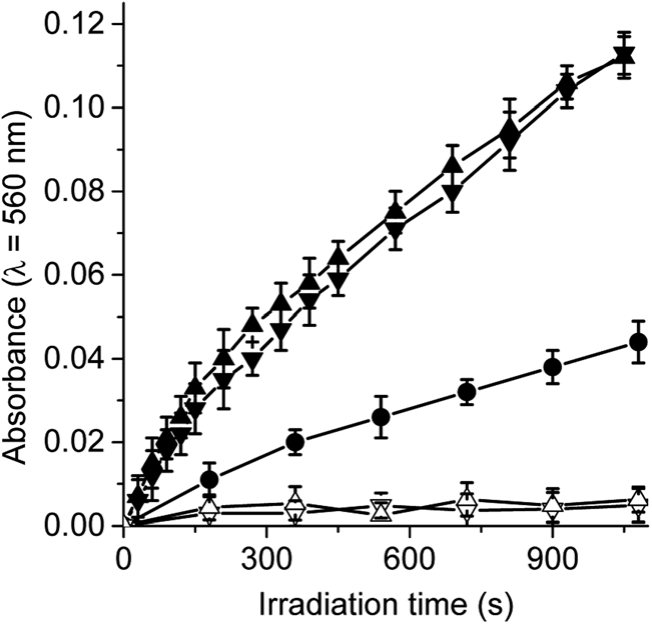
Time course of NBT reduction as an increase in the absorption at *λ* = 560 nm in DMF/10% water. Samples contain NBT (0.2 mM), NADH (0.5 mM) and TCP-C_60_ (▲) (1.2 μM) or TCP-C_60_^4+^ (▼) (1.2 μM) in DMF/10% water irradiated with light at *λ*_irr_ = 428 nm. Controls: NBT and NADH (●), TCP-C_60_ and NTB (△); TCP-C_60_^4+^ and NBT (▽).

Under aerobic conditions, the decomposition of NBT occurred predominantly through a type I process, indicating the formation of O_2_˙^−^.^29,30^ Thus, when solutions of dyads containing NBT and NADH were irradiated, the generation of diformazan was observed in DMF/10% water, as shown by the characteristic colour change of the reaction (Fig. S2†). The reduction of NBT to diformazan was examined following the increase in absorption at 560 nm. Fig. 3 shows the changes in the absorption as a function of time after irradiation of samples. For both dyads, reduction of NBT was negligible in the absence of NADH. Without the dyad in solution, a slight reduction of NBT was observed in presence of NADH (Fig. 3). In all cases, the presence of NADH was needed for the formation of diformazan. Therefore, an increase in the photoreduction of NBT was observed in the presence of dyad and NADH with respect to solution without the photosensitizer. These results showed that TCP-C_60_ or TCP-C_60_^4+^ in a polar homogeneous medium can form a photoinduced charge-separated state resulting in the production of O_2_˙^−^. In a previous work, the generation of O_2_˙^−^ was found with a dicationic fullerene C_60_ derivative in presence of NBT and NADH, in benzene/BHDC (0.1 M)/*W*_0_ = 10 reverse micelles.^31^ Moreover, the decomposition of NBT sensitized by 5,10,15,20-tetrakis[3-(*N*-ethyl-*N*-methylcarbazoyl)]chlorin or its analogous porphyrin was found indicating that both photosensitizers considerably produced O_2_˙^−^ in presence of NADH.^32^ Under similar conditions, the generation of O_2_˙^−^ was also observed with a complex micelle formed by zinc tetrakis(4-sulfonatophenyl)porphyrin, modified fullerene and poly(ethylene glycol)-*block*-poly(l-lysine).^28^

On the other hand, the production of O_2_(^1^Δ_g_) by these dyads was previously investigated in solvents of different polarities.^25^ An efficiently produce O_2_(^1^Δ_g_) sensitized by TCP-C_60_ (*Φ*_Δ_ = 0.56) was found in a non-polar solvent, such as toluene. However, the type II photoprocess mediated by both dyads considerably decrease (*Φ*_Δ_ = 0.01 and 0.02 for TCP-C_60_ and TCP-C_60_^4+^, respectively) in DMF. Therefore, a competitive process must be involved in this more polar solvent. This behaviour can be attributed to the formation of photoinduced charge-separated state, which can favour the generation of O_2_˙^−^ in a polar medium.

### 3.3. Photooxidation of Trp

**Fig. 4:**
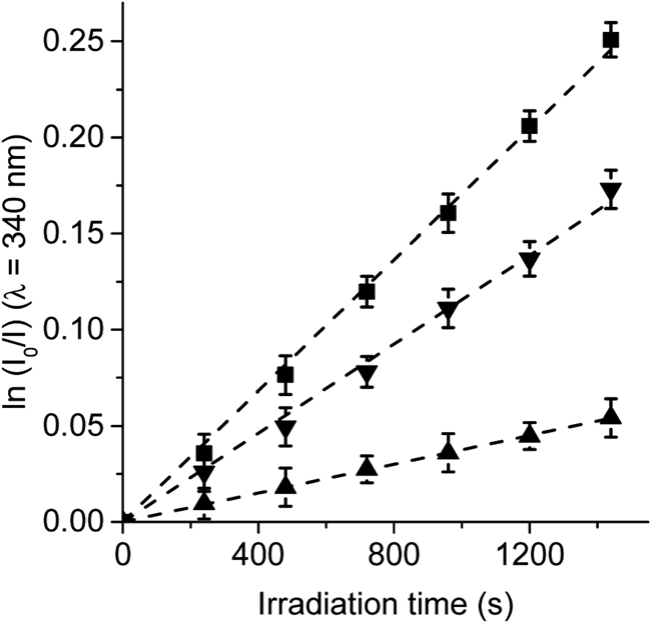
First-order plots for the photooxidation of Trp (20 μM) photosensitized by TCP (■), TCP-C_60_ (▲) and TCP-C_60_^4+^ (▼) in DMF; *λ*_irr_ = 428 nm.

Trp amino acid residues is one of the amino acids most susceptible to oxidation and can be a potential target of the ROS produced by photosensitizers in microbial cells.^33,34^ Moreover, Trp can be photooxidized by both type I and type II mechanisms.^35^ Photosensitized decomposition of Trp sensitized by TCP, TCP-C_60_ and TCP-C_60_^4+^ was evaluated by the decrease of fluorescence in DMF (Fig. S3†). As shown in Fig. 4, the photodecomposition of Trp followed first-order kinetics with respect to substrate concentration. The results of *k*^Trp^_obs_ calculated for Trp decomposition are summarized in Table 1. A higher value was found for the reaction rate sensitized by TCP in comparison with dyads. It was previously demonstrated that the TCP presents considerable photodynamic activity in DMF because it has a significant contribution of type II mechanism.^25^ In contrast, a low O_2_(^1^Δ_g_) production by dyads was observed in a polar solvent. Therefore, the decomposition of Trp mediated by dyads may be mainly due to type I mechanism of action. Moreover, the results shown in Table 1 indicate a higher value of *k*^Trp^_obs_ for the reaction photosensitized by TCP-C_60_^4+^ than TCP-C_60_. These results are not in agreement with the O_2_(^1^Δ_g_) production sensitized by these dyads.^25^ A possible interaction between these dyads and Trp could be favouring an electron transfer process in the decomposition of the amino acid. Thus, the interaction of photosensitizers singlet excited state with Trp was studied by steady-state fluorescence. Fig. S4† shows the Stern–Volmer plots of Trp at different photosensitizer concentration in DMF. Values of *K*_SV_ = 401 ± 20, 329 ± 15 and 57 ± 3 M^−1^ were obtained for TCP-C_60_, TCP-C_60_^4+^ and TCP, respectively. Taking into account a *τ*^0^ = 2.5 ns for Trp in DMF,^36^ the values of *k*_q_ (∼1 × 10^11^ s^−1^) are over diffusion limit in DMF. The ability of the fullerene C_60_ to interact with amino acids was previously calculated suggesting that the most favourable interactions of the fullerene are with arginine, leucine, and tryptophan, which is related to the backbone structure of the corresponding amino acids.^37^

**Table 1:** Kinetic parameters for the photooxidation reaction of Trp (*k*^Trp^_obs_)

Photosensitizer	Solvent	TCP	TCP-C_60_	TCP-C_60_^4+^
*k* ^Trp^_obs_ (s^−1^)^a^	DMF	(1.71 ± 0.02) × 10^−4^	(3.76 ± 0.02) × 10^−5^	(1.16 ± 0.02) × 10^−4^
*k* ^Trp^_obs_ (s^−1^)^b^	DMF	(3.34 ± 0.05) × 10^−4^	(1.15 ± 0.06) × 10^−4^	(1.70 ± 0.2) × 10^−4^
*k* ^Trp+Car^_obs_ (s^−1^)^b^^,^^c^	DMF	(2.27 ± 0.05) × 10^−4^	(1.13 ± 0.06) × 10^−4^	(1.60 ± 0.2) × 10^−4^
*k* ^Trp^_obs_ (s^−1^)^b^	DMF/2.5% water	(8.00 ± 0.4) × 10^−5^	(2.33 ± 0.09) × 10^−5^	(7.50 ± 0.2) × 10^−5^
*k* ^Trp+Az^_obs_ (s^−1^)^b^^,^^d^	DMF/2.5% water	(3.80 ± 0.2) × 10^−5^	(2.19 ± 0.08) × 10^−5^	(5.50 ± 0.2) × 10^−5^
*k* ^Trp^_obs_ (s^−1^)^b^	DMF/5% water	(6.50 ± 0.2) × 10^−5^	(1.35 ± 0.03) × 10^−5^	(3.70 ± 0.2) × 10^−5^
*k* ^Trp+Ma^_obs_ (s^−1^)^b^^,^^e^	DMF/5% water	(6.40 ± 0.2) × 10^−5^	NR	(3.60 ± 0.2) × 10^−5^

a *λ*_irr_ = 428 nm.

b *λ*_irr_ = 562 nm.

c β-Carotene.

d Sodium azide.

e d-Mannitol.

**Fig. 5:**
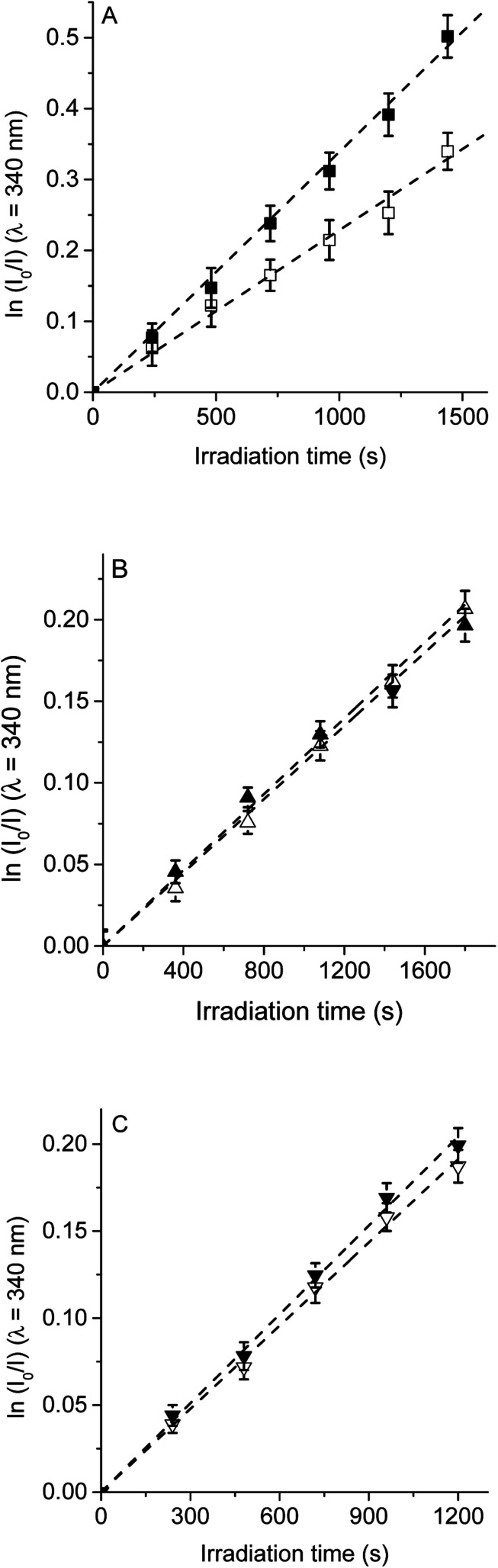
First-order plots for the photooxidation of Trp (20 μM) photosensitized by (A) TCP (■) and TCP in presence of β-carotene (□), (B) TCP-C_60_ (▲) and TCP-C_60_ in presence of β-carotene (△) and (C) TCP-C_60_^4+^ (▼) and TCP-C_60_^4+^ in presence of β-carotene (▽) in DMF; *λ*_irr_ = 562 nm.

**Fig. 6:**
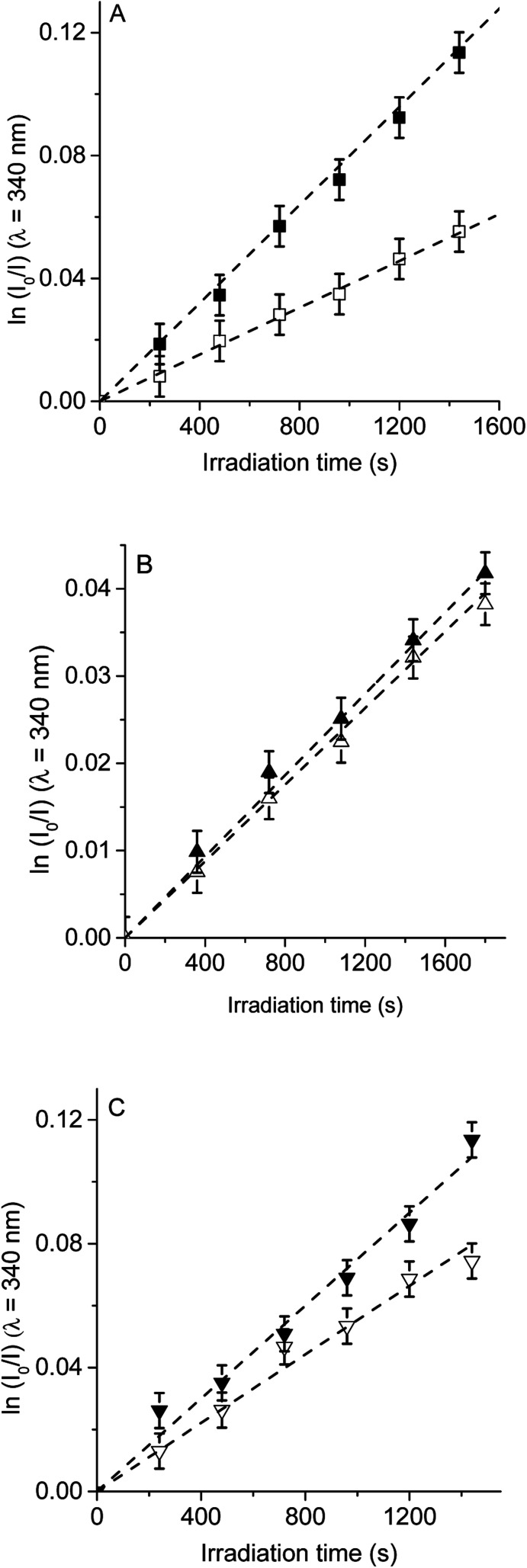
First-order plots for the photooxidation of Trp (20 μM) in photosensitized by (A) TCP (■) and TCP in presence of sodium azide (□), (B) TCP-C_60_ (▲) and TCP-C_60_ in presence of sodium azide (△) and (C) TCP-C_60_^4+^ (▼) and TCP-C_60_^4+^ in presence of sodium azide (▽) in DMF/2.5% water; *λ*_irr_ = 562 nm.

**Fig. 7:**
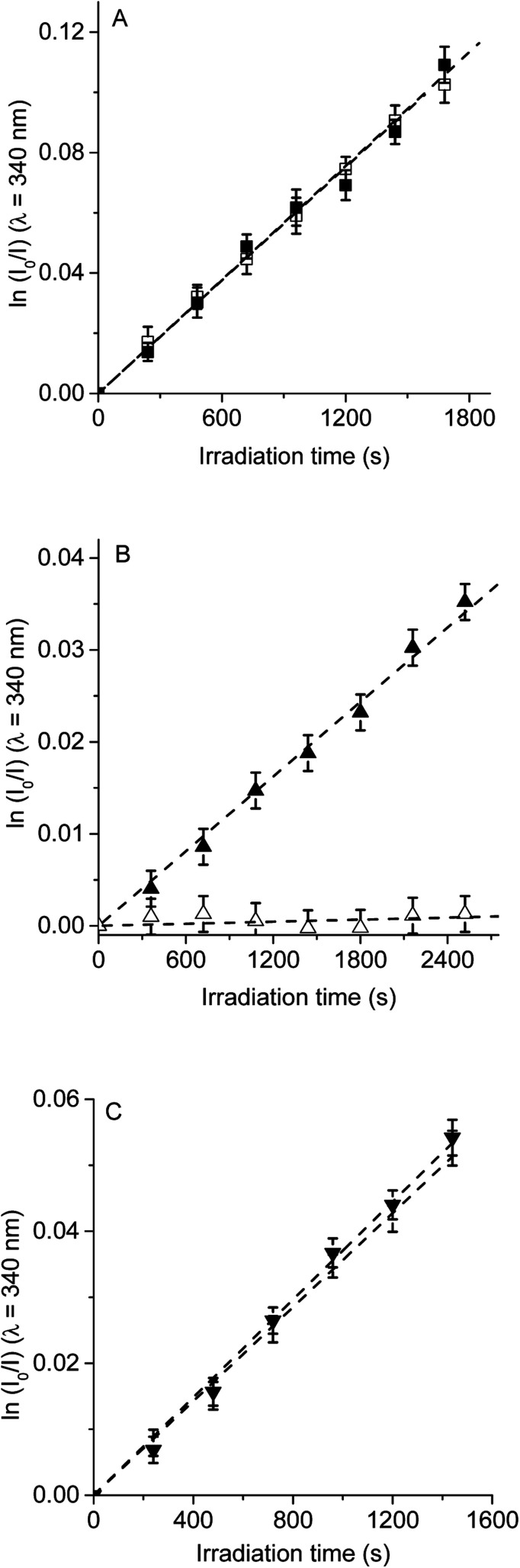
First-order plots for the photooxidation of Trp (20 μM) photosensitized by (A) TCP (■) and TCP in presence of d-mannitol (□), (B) TCP-C_60_ (▲) and TCP-C_60_ in presence of d-mannitol (△) and (C) TCP-C_60_^4+^ (▼) and TCP-C_60_^4+^ in presence of d-mannitol (▽) in DMF/5% water; *λ*_irr_ = 562 nm.

Therefore, to evaluate the main photodynamic mechanism involved in the Trp decomposition photosensitized by these dyads, experiments were carried out in presence of different ROS scavenges. The photodynamic effect on the Trp decomposition was investigated in the presence of β-carotene, sodium azide or d-mannitol. Solutions of TCP, TCP-C_60_ or TCP-C_60_^4+^ containing Trp and scavenger were irradiated with monochromatic light (*λ*_irr_ = 562 nm). This wavelength was chosen to avoid β-carotene absorption and this light was mainly used to excite the porphyrin moiety in the dyads. A low amount of water addition was needed to solubilize sodium azide and d-mannitol. The values of *k*^Trp^_obs_ in the presence and absence of scavengers were calculated from first-order kinetic plots of the Trp (Fig. 5–7) sensitized by TCP, TCP-C_60_ and TCP-C_60_^4+^. The results of *k*^Trp^_obs_ are shown in Table 1.

First, the photodynamic effect was evaluated by decomposition of Trp in the presence of β-carotene (Fig. 5). Under these conditions, β-carotene can deactivate O_2_(^1^Δ_g_) through energy transfer or by chemical reaction.^38,39^ The photodecomposition of Trp sensitized by TCP in the presence of β-carotene showed a lower value of *k*^Trp^_obs_ than in absence of β-carotene (Table 1). Since the TCP mainly produces O_2_(^1^Δ_g_), it was expected a photoprotective effect by β-carotene. However, in the presence of TCP-C_60_ or TCP-C_60_^4+^ the values of *k*^Trp^_obs_ were slight affected by the addition of β-carotene. In a polar solvent, such as DMF, dyads can form a photoinduced charge-separated state diminishing its ability to generate O_2_(^1^Δ_g_).^23^

Photodecomposition of Trp was studied in presence of sodium azide in DMF/2.5% water (Fig. 6). Azide ions can deactivate O_2_(^1^Δ_g_) and other compounds in its excited triplet state through an energy transfer.^40^ A lower value of *k*^Trp^_obs_ was obtained when for the reaction sensitized by TCP in the presence of azide ions (Table 1), according with the formation of O_2_(^1^Δ_g_). In contrast, for TCP-C_60_ and TCP-C_60_^4+^ the values of *k*^Trp^_obs_ were little affected by the addition of sodium azide, indicating a contribution of type I mechanism in Trp photooxidation process. Although the addition of β-carotene did not affect the rate of Trp photooxidation sensitized by TCP-C_60_^4+^ (Fig. 5C), the addition of sodium azide slowed it (Fig. 6C). This behavior may be due to the electrostatic interaction of the azide anions with the cationic TCP-C_60_^4+^ dyad, favoring the deactivation of the excited triplet state of the photosensitizer.

The photosensitized decomposition of Trp was also analysed in the presence of d-mannitol in DMF/5% water (Fig. 7). This compound can act as a radical scavenger.^41^ Therefore, it was used to verify the presence of type I mechanism.^23^ The value of *k*^Trp^_obs_ for TCP was not modified by the addition of d-mannitol (Table 1), which was due to TCP generates mainly O_2_(^1^Δ_g_). The photodecomposition of Trp sensitized by TCP-C_60_ in the presence of d-mannitol was negligible, according with the formation a photoinduced charge-separated state diminishing its ability to generate O_2_(^1^Δ_g_). Therefore, the addition of d-mannitol proves evidence that its primary mechanism of action was type I in this polar medium. In the case of TCP-C_60_^4+^, the presence of d-mannitol did not affect the value of *k*^Trp^_obs_. As indicated above, cationic photosensitizers may interact with Trp and produces the decomposition of the amino acid by a radical charge transfer process. Thus, the interaction between the cationic dyad and Trp avoid the effect d-mannitol on photooxidation rate. In presence of dyads, Trp can produce Trp radical, which can react with O_2_˙^−^ to form Trp hydroperoxide that can then rearrange into *N*-formylkynurenine and kynurenine.^34^

### 3.4. Photodynamic action in *S. aureus* cells

**Fig. 8:**
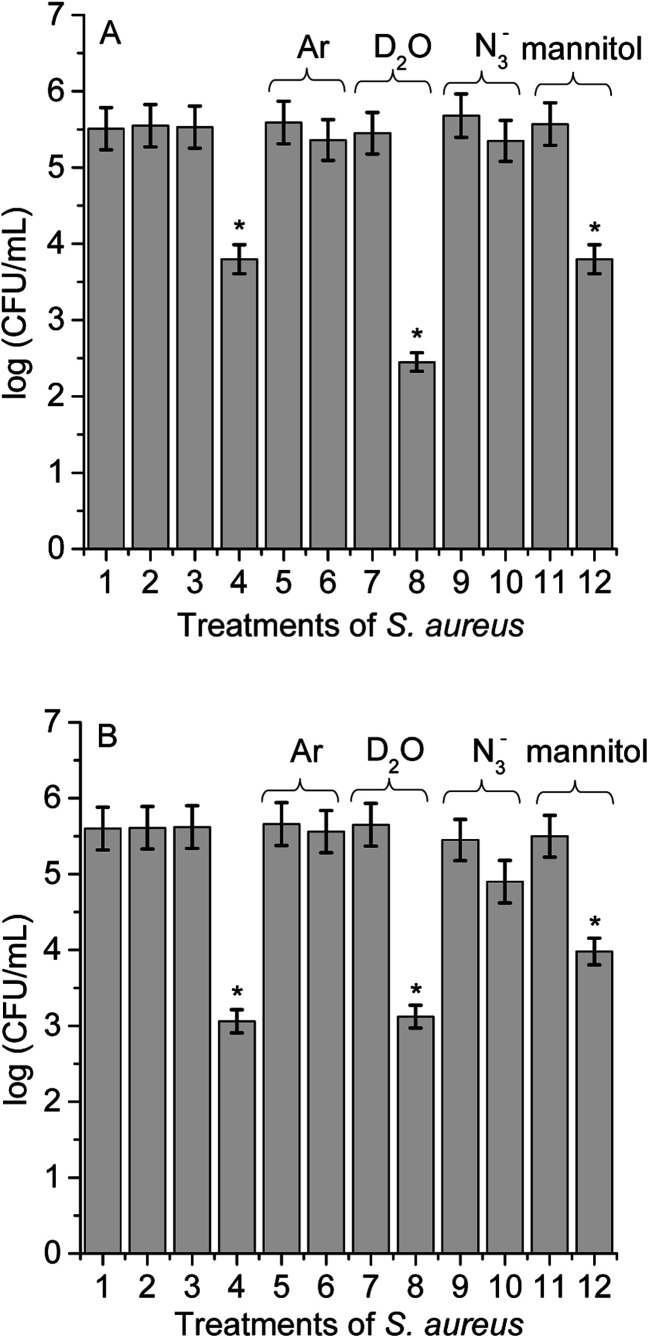
Survival of *S. aureus* cells (∼10^6^ CFU mL^−1^) incubated with 1 μM TCP-C_60_ (A) and TCP-C_60_^4+^ (B) for 30 min at 37 °C in dark and exposed to visible light for 15 min; (1) cells in dark; (2) irradiated cells; (3) cells treated with dyad in dark; (4) irradiated cells treated with dyad; (5) irradiated cells under argon; (6) irradiated cells treated with dyad under argon; (7) irradiated cells in D_2_O; (8) irradiated cells treated with dyad in D_2_O; (9) irradiated cells containing 50 mM sodium azide; (10) irradiated cells treated with dyad containing 50 mM sodium azide; (11) irradiated cells containing 50 mM d-mannitol; (12) irradiated cells treated with dyads containing 50 mM d-mannitol (*p < 0.05, compared with control).

Different experimental conditions were used to detect ROS sensitized by TCP-C_60_ or TCP-C_60_^4+^ in *S. aureus* cell suspensions. The effect of medium on the photoinactivation of *S. aureus* was investigated in an atmosphere of argon and in cell suspensions in D_2_O. Moreover, the activity of scavengers of ROS on the photoinactivation of *S. aureus* was evaluated by the addition of sodium azide or d-mannitol. In a previous work, these dyads were effective photosensitizers to inactivate *S. aureus* after irradiation with visible light.^16^ The results showed that PDI mediated by TCP-C_60_^4+^ was slight more effective than by TCP-C_60_. In particular, the cationic dyad TCP-C_60_^4+^ exhibited a photosensitizing activity producing a 4.5 log decrease of cell survival, when the cells were treated with 5 μM dyad and irradiated for 30 min with visible light. In this work, studies of PDI were performed with this Gram-positive bacterium in order to obtain information about the main photoprocesses involved in the photokilling of cells. For cells treated with 1 μM dyad and 15 min irradiation, the decrease in cell survival was 1.8 log and 2.5 log for TCP-C_60_ and TCP-C_60_^4+^, respectively (Fig. 8, line 4). Moreover, no toxicity was observed for *S. aureus* cells not treated with the dyads after 15 min irradiation (Fig. 8, line 2) or treated with 1 μM dyad in dark (Fig. 8, line 3). Therefore, these conditions were used to determine the principal mechanism involved in PDI because it allowed seeing an increase or a decrease in the photoinactivation of bacteria.

Photoinactivation of *S. aureus* cell by dyads was studied under anoxic condition. Cell viability was not modified after irradiation without dyad in an atmosphere of argon (Fig. 8, line 5) or in the presence of dyad in the dark (result not shown). Furthermore, the photoinactivation of *S. aureus* was negligible for cells incubated with TCP-C_60_ or TCP-C_60_^4+^ after 15 min irradiation (Fig. 8, line 6). Although, the presence of oxygen is essential for the generation of O_2_(^1^Δ_g_) through the type II photosensitization mechanism, oxygen also plays a major role in the type I mechanism by adding to biochemical radicals.^12^ In a type I process, the light excites the photosensitizers and it interacts with a substrate to yield radical ions by a hydrogen atom or electron transfer reaction. The majority of these radicals react with oxygen instantaneously and generate a complicated mixture of highly reactive oxygen intermediates, which can oxidize a wide variety of biomolecules. Therefore, these experiments are not decisive in establishing the predominant photoreaction process involved in the cytotoxicity. Nevertheless, this result shows that these dyads were not able to inactivate microorganisms under anoxic conditions. Similar results were found with *Streptococcus mitis* cell suspensions treated with the phthalocyanine ZnEPc^4+^ under an argon atmosphere.^42^ The loss of viability of *S. mitis* cells was highly oxygen dependent. Furthermore, other photosensitizers were reporter with the same behaviour.^23,40^

To evaluate the O_2_(^1^Δ_g_) mediated photoinactivation of microorganisms, the PDI was performed in D_2_O because it is well known that D_2_O increases the lifetime of O_2_(^1^Δ_g_) (*τ*_0_ = 68 μs in D_2_O and *τ*_0_ = 4.2 μs in water).^43^ No toxicity was detected in the presence of D_2_O under irradiation without dyad (Fig. 8, line 7) or cells treated with dyad in dark (result not shown). Irradiation of *S. aureus* in D_2_O with TCP-C_60_ produced a more pronounced reduction in cell viability (1.5 log) than in PBS cell suspensions (Fig. 8A, line 8). The values found for the non-charged dyad showed that there was a contribution of O_2_(^1^Δ_g_) in cell inactivation. However, the photocytotoxic activity induced by TCP-C_60_^4+^ was similar in D_2_O respect to PBS cell suspensions (Fig. 8B, line 8). This result with cationic dyad revealed a low contribution of type II mechanism. The different behaviours of dyads could be due to different intracellular localization of the photosensitizers in *S. aureus* cells.^23^

Moreover, in order to assess the involvement of O_2_(^1^Δ_g_) in the photoinactivation of *S. aureus* cells, experiments were carried out in the presence of sodium azide.^44^*S. aureus* cells were treated with 50 mM sodium azide and 1 μM dyad. This amount of azide ions was not toxic after 15 min with visible light (Fig. 8, line 9) or in the dark containing the dyad (result not shown). The presence of azide ions produced almost complete photoprotection of *S. aureus* cells treated with TCP-C_60_ or TCP-C_60_^4+^ and irradiated for 15 min (Fig. 8, line 10). Sodium azide is known to inhibit the photodynamic damage of O_2_(^1^Δ_g_) in bacteria cells.^23^ This is due to the fact that the azide ions can act as a quencher of O_2_(^1^Δ_g_) but also can deactivate compounds in their triplet excited state.^45^ Thus, the presence of azide ions quenched the photocytotoxic species, producing a protective effect on *S. aureus* cells.

As well, the photoinactivation of *S. aureus* mediated by 1 μM dyad was examined after incubation with 50 mM d-mannitol. This compound can be used as a scavenger of O_2_˙^−^ and HO˙ (type I reaction).^41^ The addition of 50 mM d-mannitol was not toxic to irradiated cells without dyad (Fig. 8, line 11) or containing the d-mannitol and dyad in dark (result not shown). After 15 min irradiation of treated cells, the results indicated that phototoxicity efficacy of TCP-C_60_ to inactivate *S. aureus* cells was not significantly affected (Fig. 8A, line 12). Non-charged dyad may be located in a non-polar cellular microenvironment favouring the production of O_2_(^1^Δ_g_). On the other hand, ∼1 log photoprotective effect was found for bacterial cells treated with TCP-C_60_^4+^ in presence of d-mannitol (Fig. 8B, line 12). Therefore, a contribution of type I pathway was found for *S. aureus* sensitized by cationic dyad.

Previous studies about the contribution of ROS in the photodynamic inactivation of a Gram-positive bacterium *Enterococcus hirae* by *meso*-tetra(*N*-methyl-4-pyridyl)porphyrin (TMPyP) was investigated using several scavengers.^40^ The results demonstrated that for Gram-positive bacteria, type I reactions appear to play a minor role compared to type II reactions. On the other hand, mechanistic studies on the photodynamic effect induced by fullerene C_60_ derivative on *S. aureus* cells showed that under aerobic condition the photocytotoxicity activity induced by fullerene was mediated by mainly a contribution of type II process.^23^ However, dyads TCP-C_60_ and TCP-C_60_^4+^ have a porphyrin moiety covalently bound to fullerene C_60_. Therefore, the mechanism of photodynamic action involved will depend on the polarity of the medium in which the molecules were dissolved. In a polar microenvironment, a photoinduced charge separation state may be formed, favoring a type I photoprocesses. In a non-polar medium, an energy transfer mechanism can occurs, increasing the production of O_2_(^1^Δ_g_).^16^

### 3.5. Photooxidation of DMA in *S. aureus* cells

**Fig. 9:**
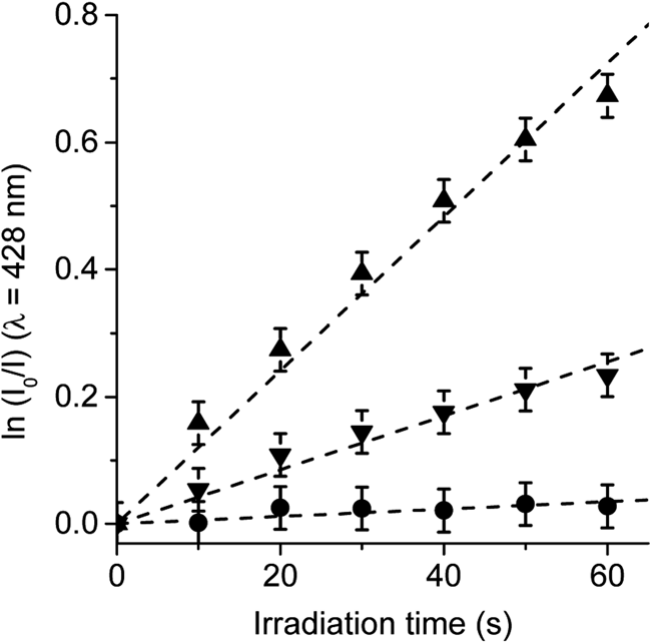
Photooxidation of DMA in *S. aureus* cells (∼10^6^ CFU mL^−1^) incubated with 10 μM DMA for 30 min followed by a washing step and treated with 1 μM TCP-C_60_ (▲) or TCP-C_60_^4+^ (▼) for 30 min at 37 °C in dark. Control: *S. aureus* cells incubated with 10 μM DMA (●); *λ*_irr_ = 455–800 nm.

In order to confirm the production of O_2_(^1^Δ_g_) by TCP-C_60_ and TCP-C_60_^4+^ dyads in *S. aureus* cells, the photooxidation of DMA was evaluated in bacterial suspensions monitoring fluorescence emission decrease (Fig. S5†). This anthracene derivative can act as photosensitizer itself producing O_2_(^1^Δ_g_) when it is irradiated.^46^ Therefore, the system was illuminated in a range (*λ*_irr_ = 455–800 nm) where DMA do not absorb. Hence, PDI of *S. aureus* was not affected by the addition of DMA. Furthermore, shorter irradiation times were used in kinetic studies, with a total time of 1 min. Considering that DMA quenches O_2_(^1^Δ_g_) by chemical reaction to form the corresponding endoperoxide, it is used as a method to evaluate the ability of dyads to produce O_2_(^1^Δ_g_) in *S. aureus* cells.^23^ The values of the observed rate constant (*k*^DMA^_obs_) were calculated from first-order kinetic plots of the DMA emission at 428 nm with time (Fig. 9). Values of *k*^DMA^_obs_ = (1.26 ± 0.06) × 10^−2^ s^−1^ and (4.18 ± 0.08) × 10^−3^ s^−1^ were obtained for TCP-C_60_ and TCP-C_60_^4+^, respectively. The decomposition rate induced by TCP-C_60_ was three times faster than that of TCP-C_60_^4+^. The results obtained in cell media showed that TCP-C_60_ can be located in a less polar cellular environment than TCP-C_60_^4+^. Therefore, TCP-C_60_ can inactivate the microbe mainly by type II mechanism of photosensitization. Similar results were found using the photosensitizer zinc(ii) 2,9,16,23-tetrakis[4-(*N*-methylpyridyloxy)]phthalocyanine in *Candida albicans* cells.^47^ On the other hand, the photooxidation of DMA mediated by *N*,*N*-dimethyl-2-[4-(3-*N*,*N*,*N*-trimethylammoniopropoxy) phenyl]fulleropyrrolidinium (DPC_60_^2+^) was studied in *S. aureus* cells.^23^ A lower production of O_2_(^1^Δ_g_) of DPC_60_^2+^ than 5,10,15,20-tetrakis(4-*N*,*N*,*N*-trimethylammoniumphenyl)porphyrin (TMAP^4+^) was found in cell suspensions. Te slower photooxidation rate of DMA mediated by DPC_60_^2+^ was attributed to a lower O_2_(^1^Δ_g_) production than TMAP^4+^ in *S. aureus* cells. However, a different intracellular localization of the photosensitizers can also affect the decomposition rate of DMA due to the short lifetime of O_2_(^1^Δ_g_) inside the cells. Among other variables, the photodynamic mechanism of action depend on the medium.^48^ Therefore, it is not possible to make direct correlations between the data obtained in solution and in microbial cells.

## 4. Conclusions

Type I and II mechanisms are present in the PDI induced by porphyrin-fullerene C_60_ dyads both in solution and in *S. aureus* cells. Studies of steady-state photolysis in the presence of an electron acceptor and an electron donor confirm that TCP-C_60_ and TCP-C_60_^4+^ dyads are capable to form a photoinduced charge-separated state in a polar medium. Furthermore, both dyads produced O_2_˙^−^ in the presence of NADH, as a biological reducing agent. Although both dyads have a very low production of O_2_(^1^Δ_g_), they sensitized the photooxidation of Trp. A slight protection in the decomposition of Trp was found in presence of β-carotene and sodium azide, which indicates a low contribution of type II photoprocess. However, the oxidation of Trp sensitized by TCP-C_60_ was negligible in solution containing d-mannitol, while the reaction rate was unaffected using TCP-C_60_^4+^. This cationic dyad electrostatically interacts with Trp and electron transfer process can occur conducing to Trp decomposition. Therefore, both dyads oxidize Trp mainly by type I mechanism of action in DMF containing water. Moreover, the present study provides knowledge about the photodynamic mechanism that takes place in the PDI of *S. aureus* cells sensitized by TCP-C_60_ and TCP-C_60_^4+^. To elucidate the oxidative processes that occur during the killing of microbial cells, the effect of the media was analyzed on cell photoinactivation. It was observed that an oxygen atmosphere was necessary for an efficient photoinactivation. The photocytotoxicity induced by TCP-C_60_^4+^ dyad in D_2_O was similar than in PBS cell suspensions. On the contrary, irradiation of bacteria treated with TCP-C_60_ in D_2_O produced a greater reduction in cell viability than in PBS cell suspensions. Photoprotection was found using sodium azide as type II scavengers. The protective effect of d-mannitol was greater for the cells in the presence of TCP-C_60_^4+^ than TCP-C_60_. Additionally, the O_2_(^1^Δ_g_) production was higher for TCP-C_60_ than TCP-C_60_^4+^ in the *S. aureus* cells. Due to the presence of positive charges on the periphery of TCP-C_60_^4+^ in comparison with non-charged TCP-C_60_, this dyad may be located in less polar cellular microenvironment. Therefore, TCP-C_60_ inactivates the *S. aureus* cells mainly by type II mechanism of photosensitization, while a greater contribution of type I pathway is involved using TCP-C_60_^4+^ as photosensitizer.

## Conflicts of interest

There are no conflicts to declare.

## Supplementary Material

RA-008-C8RA04562C-s001

RA-008-C8RA04562C-s002
