# Supplementary material for: Mechanistic insight into the photodynamic effect mediated by porphyrin-fullerene C60 dyads in solution and in Staphylococcus aureus cells
Source: RSC Adv. 2018 Jun 21;8(41):22876–86. doi: 10.1039/c8ra04562c (PMC9081455; doi:10.1039/c8ra04562c)
Supplement: RA-008-C8RA04562C-s001 [file RA-008-C8RA04562C-s001.pdf]

Electronic Supplementary Material (ESI) for RSC Advances.
This journal is © The Royal Society of Chemistry 2018

Mechanistic insight of the photodynamic effect mediated by porphyrin-fullerene C_{60} dyads in solution and *Staphylococcus aureus* cells

M. Belén Ballatore, Mariana B. Spesia, M. Elisa Milanesio and Edgardo N. Durantini*

Departamento de Química, Facultad de Ciencias Exactas, Físico-Químicas y Naturales, Universidad Nacional de Río Cuarto, Ruta Nacional 36 Km 601, X5804BYA Río Cuarto, Córdoba, Argentina.

Electronic supplementary information (ESI)

Contents

Figure S1. Spectral change observed in the steady-state photolysis of MV^{2+}.

Figure S2. Absorption spectra changes of NBT photoreduction.

Figure S3. Fluorescence emission spectra changes of Trp.

Figure S4. Fluorescence intensity of Trp.

Figure S5. Fluorescence emission spectra changes of DMA photooxidation.

Video S1. Back electron transfer process in the reduction of MV^{2+} catalyzed by oxygen, according Figure S1B.

* Corresponding author. Tel.: +54 358 4676157.

E-mail address: edurantini@exa.unrc.edu.ar (E.N. Durantini)

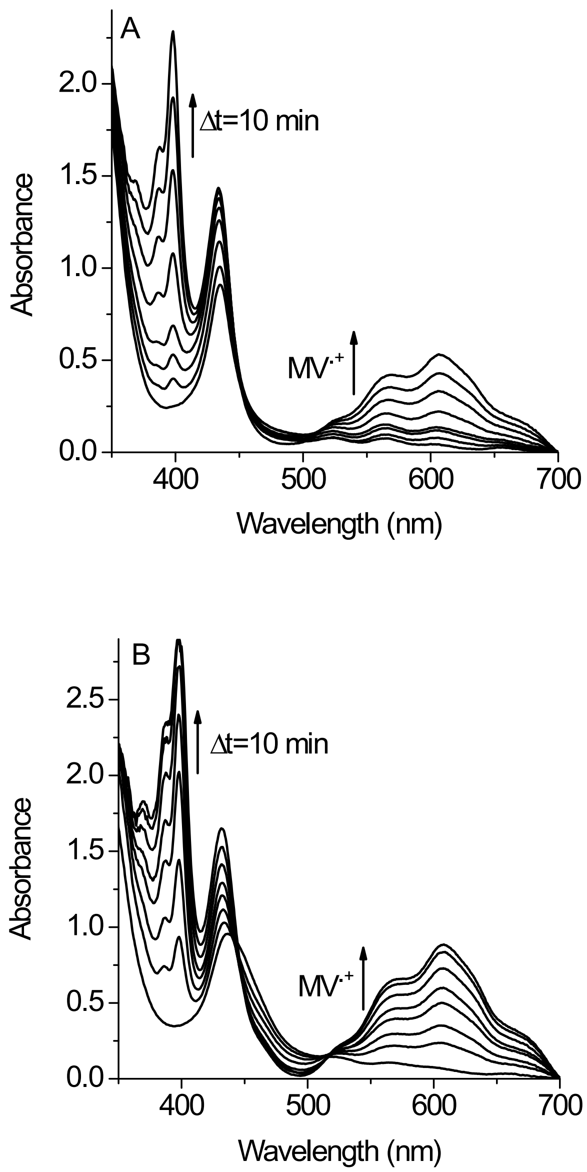

Figure S1. Spectral change observed in the steady-state photolysis of MV^{2+} (0.8 mM) sensitized by (A) TCP-C_{60} (3.0 μM) and (B) TCP-C_{60}^{4+} (3.0 μM) containing TMN (0.4 mM) after 10 min irradiation periods with visible light in DMF/5% water under an argon atmosphere, λ_{irr} = 433 nm.

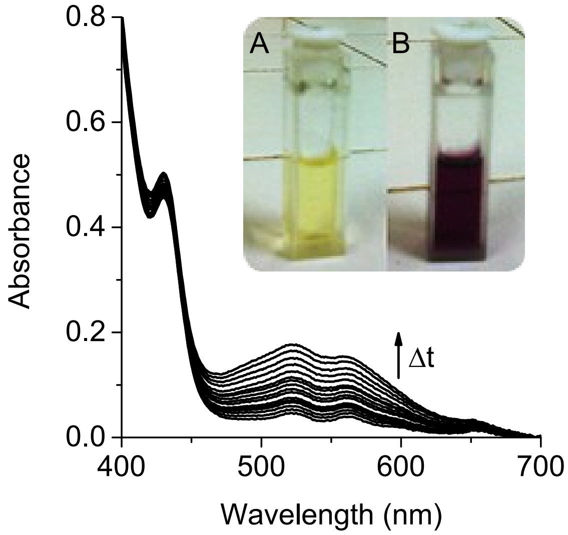

Figure S2. Absorption spectra changes of NBT photoreduction mediated by TCP-C_{60} after different irradiation times. Samples contain NBT (0.2 mM), NADH (0.5 mM) and TCP-C_{60} (1.2 μM) in DMF/10% water irradiated with visible light, λ_{irr} = 428 nm. Inset: cuvette before (A) and after (B) 18 min irradiation

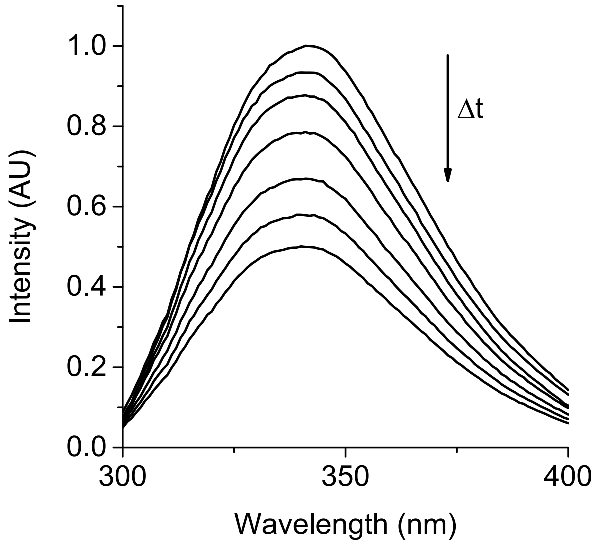

Figure S3. Fluorescence emission spectra changes of Trp photooxidation sensitized by TCP-C_{60} after different irradiation times (Δt = 240 s) in DMF; λ_{exc} = 290 nm, λ_{irr} = 428 nm.

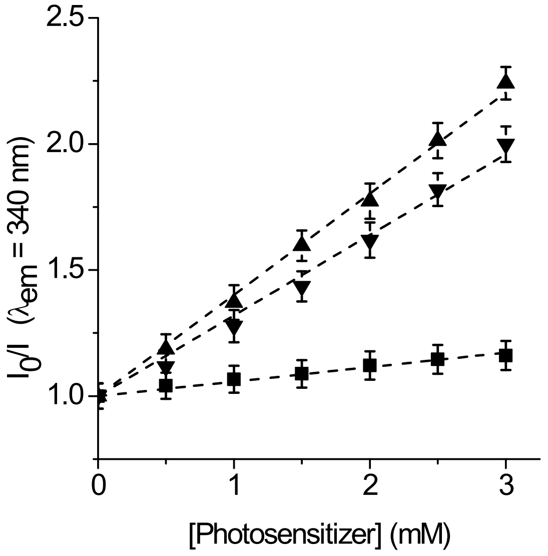

Figure S4. Fluorescence intensity of Trp (5 mM, λ_{exc}= 290 nm) in the presence of different concentration of TCP-C_{60} (◘), TCP-C_{60}^{4+} (❑) and TCP (■) in DMF.

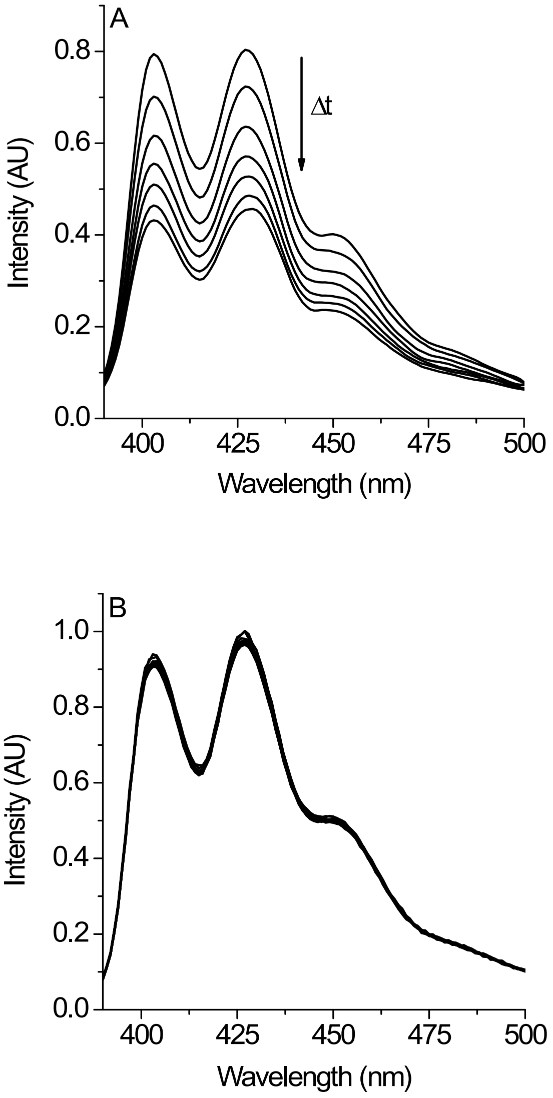

Figure S5. Fluorescence emission spectra changes of DMA photooxidation after different irradiation times ($\Delta t = 10$ s) in *S. aureus* cells (~10^6 CFU/mL) incubated with 10 μM DMA for 30 min followed by a washing step and treated with 1 μM (A) TCP-C_{60} for 30 min at 37 °C in dark and (B) control without TCP-C_{60}. λ_{irr} = 455-800 nm.
